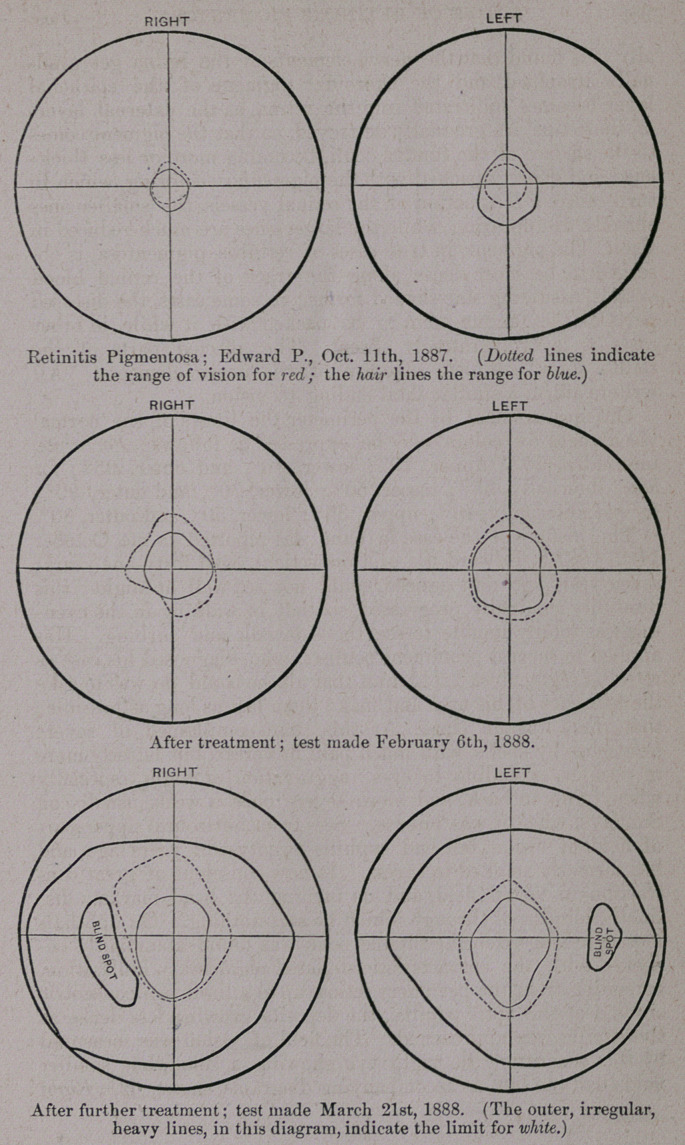# A Case of Retinitis Pigmentosa

**Published:** 1888-06

**Authors:** Frederick W. Payne

**Affiliations:** Boston


### A CASE OF RETINITIS PIGMENTOSA.

A very interesting case of retinitis pigmentosa having come to my notice, and the result of the application of the law of similars to the same having proved so exact and astounding in result, I am prompted to give you a description of the primary case, as well as a synopsis of the wonderful restoration which has progressed in regular gradation, covering a period now of a little more than six months. These astounding developments, to my mind, are more amazing from the fact that no case of pigmentary retinitis, so far as I know, has ever been reported to have improved, or even to have been arrested in its progress, much less to have been cured, although it is a disease that all oculists not infrequently encounter; on the contrary, all cases are expected to go on to loss of vision. Several classes of eye diseases considered incurable by internal medication, through allopathic authority, such as glaucoma, progressive, primary, and secondary optic nerve atrophy, retinitis apoplectica, detachment of retinæ, various forms of cataract, etc., etc., I have repeatedly seen restored to a useful condition of vision under the influence of remedies which have covered the totality of symptoms, but it has never been my fortune heretofore to have been instrumental in aiding the restoration of a case of retinitis pigmentosa.

A brief description of the disease may be useful, that those not conversant with the structural changes that take place may better understand what marvelous results are possible where the law of similars is rightly applied. The ætiology of this disease is not understood. It often appears in several members of the same family, but in the case in question there is no history of its previous appearance among his ancestry or members of his immediate family. Consanguinity is assigned as a possible cause, also hereditary syphilis. This patient's parents were not connected by ties of blood. The progress of the disease is slow, the first evidence of trouble being hemeralopia, *i. e.*, imperfect vision in a subdued light, so that night blindness becomes the most prominent objective symptom the patient recognizes. In a longer or shorter time he becomes conscious that the area of the visual field is becoming contracted, so that he must turn his head in looking so as to successively view different parts of the object looked at, especially so of any considerable superficial area; it being identically the same principle as if the normal eye was looking through a tube, hence a limit in the extent of the field; the perception for color remains as acute as formerly, being limited only as the field of vision gets more and more circumscribed; the disease attacks both eyes simultaneously, causing a limit in the visual fields to about the same extent, the contraction following an irregular concentric course, slowly advancing toward the centre, obliterating peripherally a prescribed area, till at about the age of fifty to sixty it ends in complete blindness. The night blindness is a very prominent symptom, the disability beginning each night as evening approaches, or when exposed to a dimly lighted room, so that the patient becomes entirely helpless so far as his acuteness of vision is concerned, being unable to go about alone in the evening, although his visual acuity is usually good in bright daylight. The contraction of the area of the field is noticed in any light, and gradually progresses; even where the limit of the field of vision is reduced to a few inches patients may even be able to read fine type, although unable to go about alone. Histologically it is found that the nerve elements of the retina get gradually atrophied, and the choroidal pigment of the epithelial layer becomes infiltrated into the retina, as the external layers of the retina are gradually destroyed, so that the pigment comes to the surface of the fundus oculi, becoming more or less thickened and densely packed with the pigmentary coloring, which in turn causes a contraction of the retinal vessels, the smaller ones entirely disappearing, while the larger ones are much reduced in size. The pigment, in true cases of retinitis pigmentosa, is observed to be more dense along the track of the retinal blood vessels, assuming star-shaped forms; in some cases the diseased parts of the fundus seem to be packed with it while in other cases it is comparatively absent. The affected parts of the retina show complete atrophy of the nerve elements. All writers affirm a similar fatal ending to vision.

On measurement by the perimeter the limits of the normal visual field for colors may be expressed as follows: *For white*, internally, 70°; upper, 70°; lower, 80°, and outer, 95°; *for blue*, internally, 55°; upper, 50°; lower, 70°, and outer, 90°; *for red*, internally, 40°; upper, 35°; lower, 50°, and outer, 80°.

The history of the case in hand was reported to me October 11th, 1887. Edward P., of Connecticut, aged forty-one years; seven years ago first noticed could not see well at night; this disability gradually progressed, so that in walking in the evening was totally unable to see the sidewalk and curbing. Has applied to several prominent oculists, who diagnosed his case as retinitis pigmentosa; told him that all he could do was to take the best care of his eyes and make them last as long as possible; that there was no cure. At this time complained of severe headaches by spells, with much pain in vertex, but latterly more in occiput, extending to eyes; aggravation at night, especially when lying on back, and more severe once a week, usually on Sundays, when it was intense. Sees from horizontal upper part of field of vision, but had syphilis twenty-odd years ago, and has formerly smoked to excess. Is now conscious of great contraction in visual field, and on turning the head marks a distinctly blind spot through which he sees nothing. On ophthalmoscopic inspection the fundus oculi was found abundantly bedecked with the characteristic stellated pigmentary infiltration, extending from the periphery retinæ up to a limit in a concentric circle near the optic papillæ, the deposits growing less dense as the papilla was approached. The field of vision was measured by the perimeter, the right eye showing a somewhat smaller field than the left, as accompanying diagrams show, viz.: *Right*

Retinitis Pigmentosa; Edward P., Oct. 11th, 1887. (*Dotted* lines indicate the range of vision for *red;* the *hair* lines the range for *blue.*)

After treatment; test made February 6th, 1888.

After further treatment; test made March 21st, 1888. (The outer, irregular, heavy lines, in this diagram, indicate the limit for *white.*)

*eye, for blue*, outer, 10° ; upper, 5° ; lower, 10°, and inner, 10° ; *for red*, in right eye, outer, 10° ; upper, 10° ; lower, 8°, and inner, 10°. *Left eye, for blue*, outer, 15° ; lower, 20° ; upper, 15°, and inner, 15° ; *left eye, for red*, outer, upper, lower, and inner, 10° each, making a circular space in its perception for red. Prescribed *Arum.*$^{200}$, four doses, to be taken four days apart, and Sacc. lact. in the interim. On account of the character of the symptoms being so imperfectly defined that I could scarcely more than generalize, I was led to the selection of *Arum.*, as it covered the symptoms referrable to the limit and position of the diseased visual field better than any other known remedy, as well as the nightly and periodical aggravation connected therewith, feeling that later developments might point with more definite indications to the selection of the simillimum. At the end of a fortnight he reported vision the same, but thought his headaches not so severe; continued Sacc. lact. for another fortnight. On November 11th reported headaches worse. Found him astigmatic in right eye ; selected glasses for distance, viz. : Right, —0.25, s. cyl. —0.50, ax. 150° ; left, 0 ; glasses for reading, viz. : Right + 1.25, s. cyl. —0.50, ax. 150° ; left, +1.50, s. Headache more in occiput, extending to eyes, with general blurring of vision while headache lasts; sees a single spark floating before him, more noticeable during a headache. Eyes not at all sensitive to the stimulus of light; continued same remedy, giving one dose. On January 4th, reported burning of feet at night ; must put them out of bed ; marked sinking and weakness at stomach daily, about eleven A. M. ; headaches more pronounced and regular, coming very severely once a week, on Sundays. Prescribed *Sulphur*$^{cm}$, two doses, to be followed by Sacc. lact. ; in three weeks thereafter reported an entire relief from headaches, and the stomach symptoms were much improved, as well as a relief to nightly burning of feet; vision seems brighter, *i. e.*, what he does see comes out clearer. February 6th, 1888, gave one dose of Sulph.$^{cm}$ on his tongue at office. Field of vision was again mapped out, when a marked gain was perceptible ; gets along better at night, and once, on a moonlight night, has driven a horse and carriage alone, being able to keep to the highway and avoid passing objects, a thing he has heretofore been unable to do for years. The present size of visual field for *red*, which, strange to say, is now larger than that for *blue*, a condition contrary to what usually does occur, in left eye, is, upper, 30° ; outer, 25° ; lower, 28°, and inner, 20° ; *for blue*, in left eye, is, upper, 20° ; outer, 25° ; lower, 20°, and inner, 20° ; field *for red*, in right eye, is, upper, 30° ; outer, 25° ; lower, 20°, and inner, 20° ; *for blue*, in right eye is, upper, 20° ; outer, 15° ; lower, 15°, and inner, 20° ; showing the extent of the field in each eye as being almost double for red and blue over the measurements taken at first. On March 21st, again measured the visual field ; found it still extending its limits, having about doubled its extent since the last measurements were made. Feels well ; good appetite ; no headache. When walking in the evening sees the curbing of sidewalks and gets along comfortably, a fact which his friend who was walking with him strongly commented upon. The field as now measured shows, *for red*, in left eye, upper, 30° ; outer, 25° ; lower, 45° ; inner, 30° ; *for blue*, in left eye, upper, 20° ; outer, 20° ; lower, 40°, and inner, 30° ; right eye, for *blue*, upper, 20° ; outer, 20° ; lower, 35° ; inner, 17° ; for *red*, in right eye, upper, 47° ; outer, 27° ; lower, 35°, and inner, 32°. The perception for white is now almost as large as the normal field, viz. : in left eye, upper, 60° ; outer, 85° ; lower, 75°, and inner, 70°; in right eye, upper, 55° ; outer, 85° ; lower, 75°, and inner, 70° ; the blind spots in each eye are oval, longer vertically, occupy the outer fields in right eye to a distance horizontally of 25°, and vertically, slightly obliquely, 75° ; in left eye it is smaller, occupying, horizontally, about 20°, and vertically, about 40°. Ophthalmoscopic appearances show fully as extended pigmentary deposits, but their brilliancy has markedly decreased, their sharply defined outline growing less distinct. He is still a patient, but everything points to a reasonable restoration of useful vision. The result thus far shows what change can be wrought in diseased tissues where the totality of the constitutional symptoms can be covered with the indicated remedy. Mr. P. writes me that his improvement in vision is remarkable, and he shall be "only too glad to personate the first cure of retinitis pigmentosa."

FREDERICK W. PAYNE, M. D.

BOSTON.

## Figures and Tables

**Figure f1:**